# 

**Published:** 1970-04

**Authors:** 


Bristol Medico-Chirurgical Journal Vol. 85 (ii) No. 314

### CONTENTS

What Price Screening ? — I ... ... ... ... .. ... 31

A. J. Rowland

What Price Screening ? — II ... ... ... ... ... ... 37

N. A. Dent

The Old Private Lunatic Asylum at Fishponds ... ... ... ... 41
H. Temple Phillips

A Neuropsychiatric View of Insomnia ... ... ... ... ... 45

P. K. G. Warren

Otology in the South-West ... ... ... ... ... ... 49

W. H. Bradbeer

A Visit to the Soviet Red Cross ... ... ... ... ... ... 53

D. W. Wright

The Medical Library ... ... ... ... ... ... ... 40

Obituaries ... ... ... ... ... ... ... ... ... 56

Wisdom from the Past ... ... ... ... ... ... ... 58

Society reports ... ... ... ... ... ... ... ... 60

Notes and News ... ... ... ... ... ... ... ... 62

F. Bailey & Son Ltd., Dursley, Glos.

